# Changes in Size and Interpretation of Parameter Estimates in Within-Person Models in the Presence of Time-Invariant and Time-Varying Covariates

**DOI:** 10.3389/fpsyg.2021.666928

**Published:** 2021-09-01

**Authors:** Marcus Mund, Matthew D. Johnson, Steffen Nestler

**Affiliations:** ^1^Institut für Psychologie, Friedrich-Schiller-Universität Jena, Jena, Germany; ^2^Department of Human Ecology, University of Alberta, Edmonton, AB, Canada; ^3^Institut für Psychologie, Münster University, Münster, Germany

**Keywords:** time-varying covariates, reciprocal effects, random-intercept cross-lagged panel model, autoregressive latent trajectory model with structured residuals, cross-lagged panel model

## Abstract

For several decades, cross-lagged panel models (CLPM) have been the dominant statistical model in relationship research for investigating reciprocal associations between two (or more) constructs over time. However, recent methodological research has questioned the frequent usage of the CLPM because, amongst other things, the model commingles within-person associations with between-person associations, while most developmental research questions pertain to within-person processes. Furthermore, the model presumes that there are no third variables that confound the relationships between the longitudinally assessed variables. Therefore, the usage of alternative models such as the Random-Intercept Cross-Lagged Panel Model (RI-CLPM) or the Latent Curve Model with Structured Residuals (LCM-SR) has been suggested. These models separate between-person from within-person variation and they also control for time constant covariates. However, there might also be third variables that are not stable but rather change across time and that can confound the relationships between the variables studied in these models. In the present article, we explain the differences between the two types of confounders and investigate how they affect the parameter estimates of within-person models such as the RI-CLPM and the LCM-SR.

## 1. Introduction

Question about reciprocal influences—how two or more constructs influence each other over time—are at the core of many scientific disciplines. For instance, researchers have investigated the reciprocal associations between childhood aggression and parental spanking (Berry and Willoughby, 2017), mental health and the working environment (De Lange et al., 2004), alcohol consumption and partner violence (Martino et al., 2005), community participation and psychological empowerment (Christens et al., 2011), and school climate and school academic performance (Benbenishty et al., 2016), to name just a few examples.

The most popular model for investigating reciprocal influences over time is the Cross-Lagged Panel Model (for an overview, see Biesanz, 2012). With the CLPM, it is possible to estimate the prospective effects of a variable *X* (*Y*) measured at time point *T* on variable *Y* (*X*) measured at time point *T*+1 (cross-lagged effect), while controlling for the temporal stability of both *X* and *Y* (autoregressive effect; Hertzog and Nesselroade, 2003; Biesanz, 2012). As such, the CLPM is a valuable and powerful research tool, capable of addressing a variety of interesting and important questions. However, it has also been criticized for various reasons (for overviews, see Rogosa, 1980; Allison, 2009; Hamaker et al., 2015; Berry and Willoughby, 2017). One of the major criticisms raised against the CLPM is that it assumes all individuals to vary around a common group mean in each of the included variables. However, individuals can differ in the level they vary around over time, and when such between- person differences are present in at least one of the included variables, the coefficients estimated in the CLPM are a blend of within- and between-person effects. Thus, using the CLPM can increase the risk of false interpretations and erroneous conclusions (Hamaker et al., 2015; Berry and Willoughby, 2017).

Several alternative statistical models have been developed in recent years to address this issue with the CLPM (for direct comparisons between the CLPM and alternative models, see Hounkpatin et al., 2018; Mund and Nestler, 2019; Orth et al., 2021). In the present study, we will focus on two of these models: the Random-Intercept Cross-Lagged Panel Model (RI-CLPM; Hamaker et al., 2015) and the Latent Curve Model with Structured Residuals (LCM-SR; Curran et al., 2014; Berry and Willoughby, 2017). Both the RI-CLPM and the LCM-SR explicitly take into account stable between-person differences so that their autoregressive and cross-lagged paths exclusively pertain to within-person associations (Curran et al., 2014; Hamaker et al., 2015; Berry and Willoughby, 2017).

In addition to the disaggregation of within-person and between-person effects, the RI-CLPM and the LCM-SR implicitly control for the influence of any third variable that does not change across time (e.g., the gender of participants). Thus, the within-person coefficient estimates are not affected by time-invariant covariates, either measured or *not* (Usami et al., 2019). The critical assumption, however, is that the influence of the time-invariant covariates is constant at all measurement occasions. Furthermore, there might also be time-varying covariates. Similar to the CLPM, neither the RI-CLPM nor the LCM-SR control for the effect of such variables.

The aim of the present article is to better understand the influence of time-invariant covariates (with constant or non-constant influence) and time-varying confounders on the estimates in RI-CLPM and LCM-SR. To this end, we provide a brief overview of the CLPM, the RI-CLPM, and the LCM-SR by introducing their basic features and the interpretation of the results obtained with these models. We then explain what is meant by time-invariant and time-varying covariates. Finally, we will explore the effects of (not) modeling time-varying covariates with an empirical illustration on the interplay between life satisfaction and income in a large dataset. To enable researchers to reproduce and adapt our approach to their own research questions, we provide the scripts for the R package lavaan (Rosseel, 2012) that we have used for the analysis on the Open Science Framework (https://osf.io/8mvu5/).

## 2. The Interplay Between Life Satisfaction and Income

For decades, there has been a vivid debate in various scientific fields including psychology, sociology, and economics how life satisfaction is related to income. This question is important to understand the determinants of individual well-being (Diener, 1984) and might also provide useful starting points for policy interventions including the implementation and evaluation of measures such as installing a minimum wage (Frijters et al., 2004; Ahmat et al., 2019). For instance, if there is a mutual interplay between income and satisfaction, a minimum wage might lead to increases in life satisfaction that further makes employees more productive, thus retroacting on income levels.

Across several studies, a robust correlation between life satisfaction and income has been reported (for reviews, see Diener, 1984; Diener and Biswas-Diener, 2002). This correlation has mostly been interpreted in the sense that life satisfaction is influenced by income; this line of reasoning has been supported by studies showing that changes in income are accompanied by changes in life satisfaction (Schyns, 2001; Frijters et al., 2004; Graham et al., 2004). However, it has also been theorized that life satisfaction might influence income levels (for a review, see Lyubomirsky et al., 2005). This might be due to more satisfied individual having the capacity to expand their achievements and to approach new goals (Lyubomirsky et al., 2005). Similarly, life satisfaction has been found to be associated with a personality profile that is correlated with better job performance (e.g., self-esteem, trust, agreeableness, emotional stability, hardiness DeNeve and Cooper, 1998). Self-esteem, for example, has been found to predict higher income over several years in several large-scale studies (Orth et al., 2012). Furthermore, across three large samples, Luhmann et al. (2013) have found that higher life satisfaction is associated with a decreased risk of becoming unemployed or changing jobs. In line with these findings, several studies found that life satisfaction is directly related to concurrent and future income levels (Diener et al., 2002; Graham et al., 2004; De Neve and Oswald, 2012).

Despite this large body of literature, in a review on determinants and consequences of life satisfaction, Dolan et al. (2008) noted that the findings on the role of income are controversial. Dolan et al. (2008) identified the question of directionality as one source of ambiguity, that is whether life satisfaction influences income (Diener et al., 2002; Graham et al., 2004; De Neve and Oswald, 2012) or whether income influences life satisfaction (Schyns, 2001; Frijters et al., 2004; Graham et al., 2004; Diener et al., 2010; Kahneman and Deaton, 2010). The support for a reciprocal relationship between the two constructs (Marks and Fleming, 1999; Schyns, 2001) is rather indirect, as this interplay has not been tested formally with a CLPM or a similar model.

In the following, we will examine the prospective reciprocal relationship between life satisfaction and income using a CLPM, a RI-CLPM, and an LCM-SR. In a next step, we will incorporate time-constant (i.e., gender) and time-varying covariates (i.e., self-esteem) to investigate the consequences of (not) including such variables for the parameter estimates. Before we turn to this empirical examination, we briefly describe the three statistical approaches and explain the differences between time-invariant (TIC) and time-varying covariates (TVC).

## 3. Prominent Models for Assessing Reciprocal Influences

### 3.1. Cross-Lagged Panel Model

**Figure 1:**
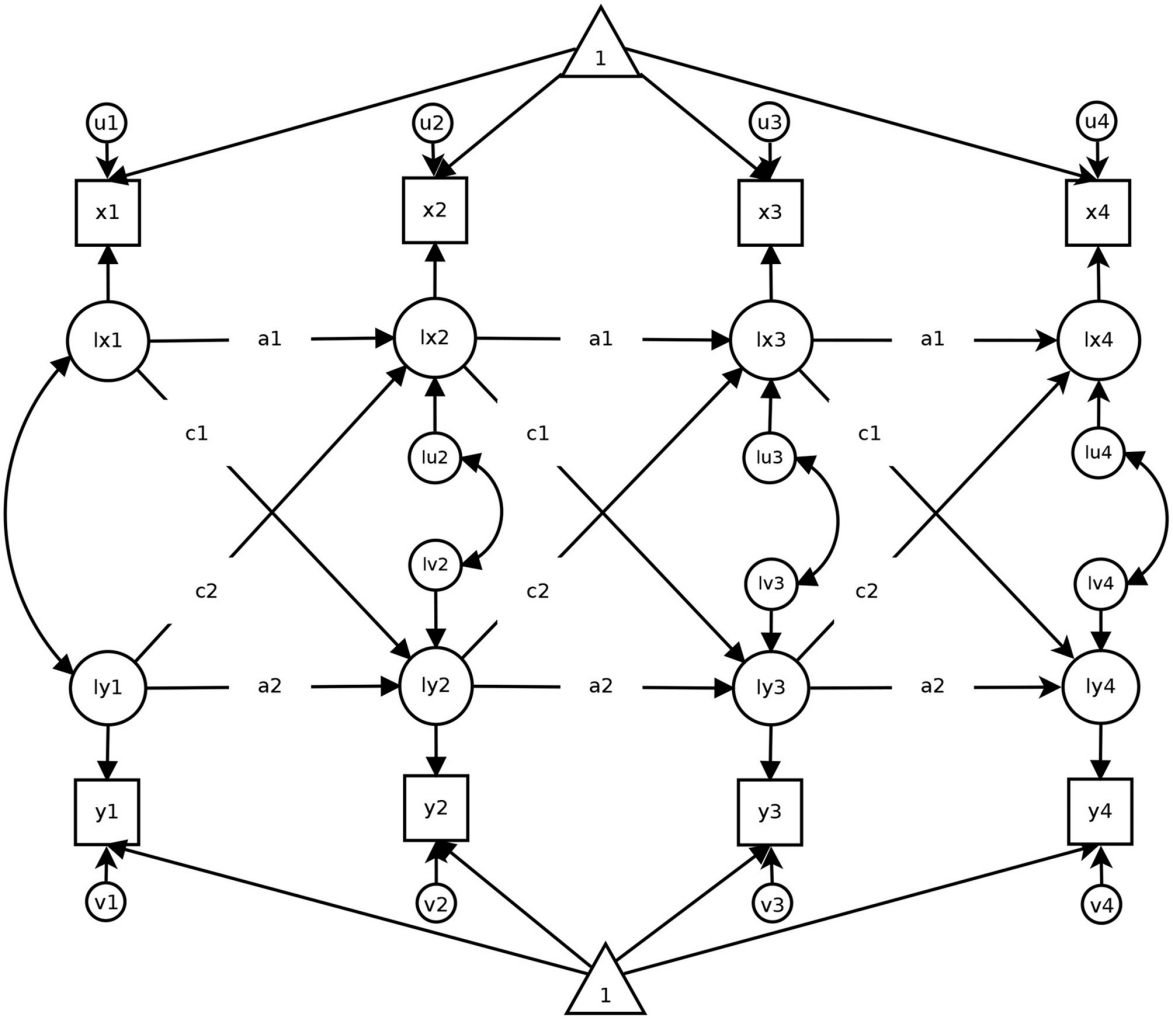
A Cross-Lagged Panel Model with four measurement occasions. Squares represent observed variables (e.g., test scores), circles indicate latent variables. Triangles refer to intercepts. Directional arrows indicate regressions, double-headed arrows indicate correlations. Equal path labels (e.g., *a*1) that the respective path was constrained to be equal across time. The figure has been published in Mund and Nestler (2019) under a CC-BY 4.0 license and is available at https://osf.io/sjph7/.

The Cross-Lagged Panel Model (CLPM) is the most widely applied model when it comes to examining the reciprocal influences between two (or more) constructs. Figure 1 displays a bivariate CLPM with four measurement occasions. The CLPM provides two key parameters: First, the autoregressive paths (*a*1 and *a*2 in Figure 1) indicate to what extent the rank order of individuals remains stable over time for variables *x* (e.g., life satisfaction) and *y* (income), respectively. Second, the cross-lagged paths (*c*1 and *c*2 in Figure 1) contain information on the strength of the reciprocal influences between *x* and *y* over time. Using our running example on life satisfaction and income, the path *c*1 (*c*2) indicates to what extent scores on life satisfaction (income) at time point *T* are prospectively associated with scores on income (life satisfaction) at the subsequent time point *T*+1. The autoregressive paths and the cross-lagged parameters are often interpreted in terms of residualized (or relative) change (Hertzog and Nesselroade, 2003; Biesanz, 2012; Hounkpatin et al., 2018; Orth et al., 2021). This means that, while the autoregressive paths indicate the stability of the rank- order, the cross-lagged paths indicate to what extent one variable is associated with prospective changes in the rank-order of the other. In the figures displaying the models and in our empirical illustration, we assume stationarity (Kenny, 1979). That is, we assume that the extent of reciprocity between life satisfaction and income does not change over time. We make this assumption to facilitate the interpretation of the results, but stationarity is not a precondition for estimating the CLPM and the alternative models discussed later.

Despite its widespread use, several authors have highlighted some potential weaknesses of the CLPM (for overviews, see Rogosa, 1980; Allison, 2009; Hamaker et al., 2015; Berry and Willoughby, 2017). One criticism is that the CLPM does not take into account stable between-person differences. This means that all individuals are assumed to vary around a common mean in *x* and *y*, respectively. However, in many cases, individuals fluctuate around a person-specific mean that is higher for some individuals than for others. For example, when considering life satisfaction, some individuals might always be more satisfied than others, relatively independent of current circumstances or external influences (Diener et al., 2006; Lucas, 2007). When such stable between-person differences are present in at least one variable, they affect the estimates of the autoregressive and cross-lagged paths and, as a consequence, might increase the probability of spurious findings (for empirical demonstrations, see Hamaker et al., 2015; Berry and Willoughby, 2017; Mund and Nestler, 2019).

### 3.2. Random-Intercept Cross-Lagged Panel Model

**Figure 2:**
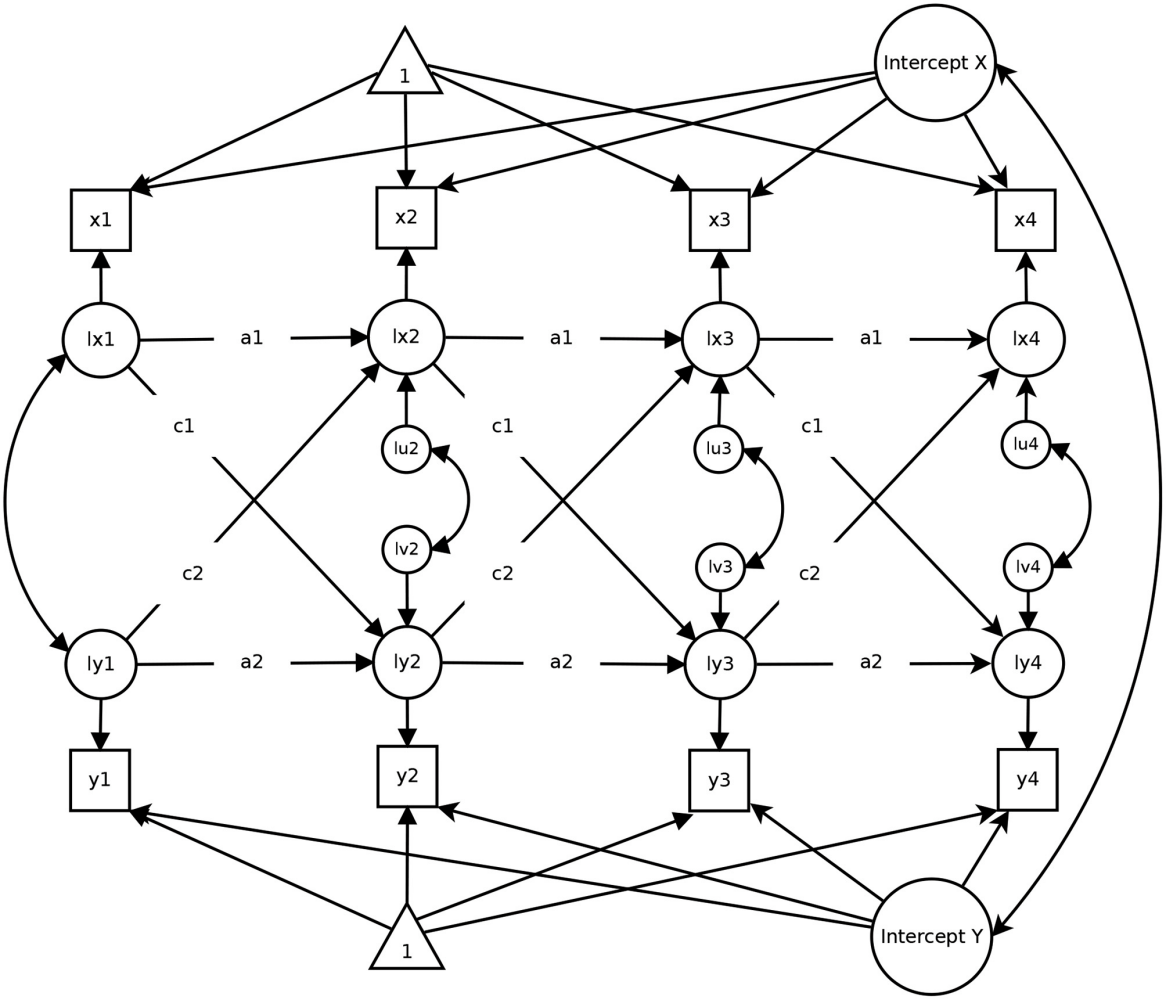
A Random-Intercept Cross-Lagged Panel Model with four measurement occasions. The figure has been published in Mund and Nestler (2019) under a CC-BY 4.0 license and is available at https://osf.io/sjph7/.

The Random-Intercept Cross-Lagged Panel Model (RI-CLPM; Hamaker et al., 2015) has been developed to take into account the stable between-person differences that are neglected in the classical CLPM. Specifically, the RI-CLPM assumes that each individual has their specific, rather stable mean on any given variable around which they fluctuate over time. These stable between-person differences are considered by modeling a latent intercept factor for each of the involved variables (see Figure 2 for an illustration). With regard to our running example, the random intercept in life satisfaction reflects the notion that some individuals are always more satisfied than others and, thus, closely resembles person-specific set points (Diener et al., 2006).

Through the specification of the random intercept factors, a person-mean centering is applied to the data. Thus, all differences between individuals that are stable (e.g., gender, ethnicity) and have a constant influence on the key variables in the model (i.e., life satisfaction and income in our example), are statistically adjusted for—even variables that have not been measured are taken into account by this approach (Allison, 2009). Through this separation of stable between-person differences from within-person differences, the RI-CLPM allows to estimate pure within-person autoregressive and cross-lagged parameters. This feature comes along with a slightly different interpretation of the estimated effects. In the RI-CLPM, the autoregressive paths *a*1 and *a*2 contain information on the within-person stability of the involved variables. Note that these paths in the classical CLPM pertain to the stability of the rank order of individuals, which is a between-person indicator (Mund et al., 2018). Likewise, the cross-lagged effects *c*1 and *c*2 in the RI-CLPM pertain to within-person associations, such that *c*1 (*c*2) indicates how strongly a deviation from the person-specific mean in, for example, life satisfaction (income) at time point *T* is associated with deviations above or below the person-specific mean in income (life satisfaction) at the subsequent time point *T*+1, controlling for previous deviations from the person-specific mean in each variable (Hamaker et al., 2015). Finally, the within-time error correlations indicate the association between the within-person residuals of *x* and *y*. As a consequence of the shift toward within-person associations, the results of the RI-CLPM can differ markedly from results obtained with the CLPM (Hounkpatin et al., 2018; Mund and Nestler, 2019; Orth et al., 2021).

### 3.3. Latent Curve Model With Structured Residuals

**Figure 3:**
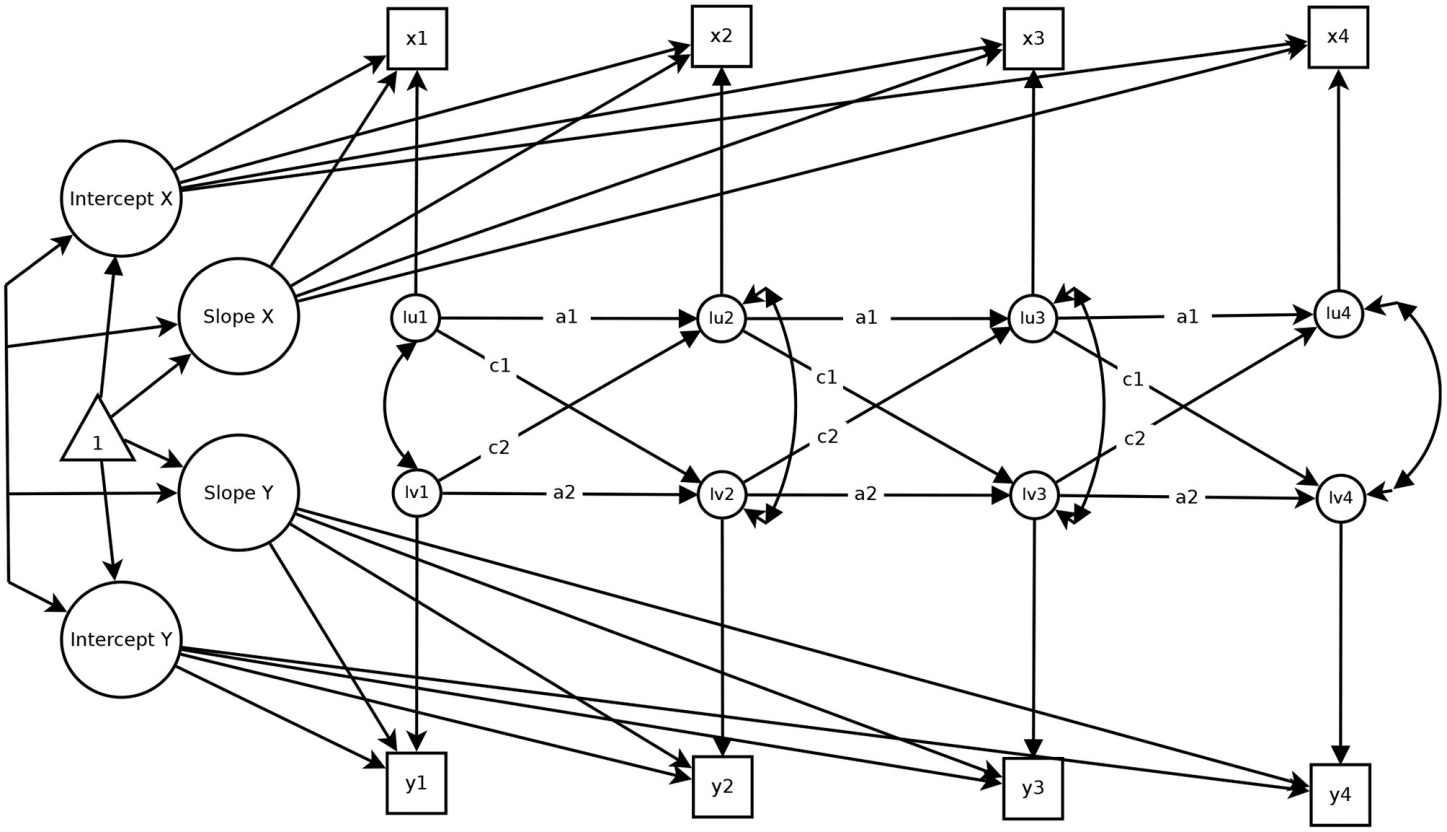
A Latent Curve Model with Structured Residuals over four measurement occasions. Figure is available under a CC-BY 4.0 license at https://osf.io/rv7xp/.

The Latent Curve Model with Structured Residuals (LCM-SR; Curran et al., 2014) for four measurement occasions is displayed in Figure 3. The LCM-SR consists of two parts: A latent growth model (LGM) and a part resembling features of a cross-lagged panel model. As in classical LGM (Bollen and Curran, 2006), the LGM portion of the LCM-SR serves to capture stable between-person differences in the levels (i.e., the latent intercept factors in Figure 3) and the individual development (i.e., the latent slope factors in Figure 3) of the included variables. Note that the growth curve can take on any functional form (Bollen and Curran, 2006; Ram and Grimm, 2007); finding the best fitting growth curve for each variable is already a crucial step in implementing the LCM-SR (Curran et al., 2014). Just like in classical LGM (Bollen and Curran, 2006), the variance of the intercepts captures between-person differences in the initial levels of the included variables. The mean and the variance of the slopes reflect the average developmental trends over time (e.g., average decreases or increases over time) and between-person differences in this development (e.g., some individuals decrease more strongly than others), respectively.

The CLPM portion of the LCM-SR is defined through the autoregressive and cross-lagged relationships between the residuals. This part of the model contains information on the pure within-person associations between *x* and *y* over time. Specifically, the residuals in the LCM-SR reflect time point specific deviations from the person-specific mean and the person-specific growth curve. Thus, the autoregressive paths *a*1 and *a*2 in Figure 3 indicate how strongly within-person deviations from the person-specific growth curve in life satisfaction (income) at time point *T* are associated with within-person deviations from the person-specific growth curve of life satisfaction (income) at the subsequent time point *T*+1. Statistically significant and strong autoregressive parameters indicate that the deviation from the person-specific curve is relatively enduring (between two measurement occasions, at least), whereas a non-significant autoregressive effect indicates that a within-person deviation is not enduring and that individuals fall back to their person-specific trajectory quite quickly. Similarly, the cross-lagged effects between the residuals indicate to what extent within-person deviations from the person-specific growth curve in life satisfaction (*c*1) or income (*c*2) at time point *T* are associated with within-person deviations from the person-specific growth curve in income or life satisfaction at time point *T*+1. The interpretation of the within-person parameters of the LCM-SR is very similar to the interpretation of the respective parameters of the RI-CLPM. In fact, the parameters are numerically identical when no developmental trends are present. However, when such trends are present, the results of the two models might be different.

Before explaining how the results of the three models can be affected by time-invariant and time-varying confounders, we note that the three models differ not only in their assumptions with regard to the model-implied covariance structure but also with regard to model-implied mean structure. In case of the CLPM, one typically estimates the means of the variables at each time point (i.e., a saturated mean structure). Thus, individuals do not vary around an overall mean, but rather around time point-specific means. The RI-CLPM is also often estimated with a saturated mean structure (e.g., Hamaker et al., 2015), but can also be estimated by constraining the means to the same value over time (see Mund and Nestler, 2019). In the second case, the model more closely resembles a bivariate random intercept multilevel model, in which the random intercepts represent the person-specific deviations from the overall mean. In the first case, by contrast, the random intercepts represent the person-specific deviations from the time-point specific means (but are themselves constant over time). Finally, because the LCM-SR contains a growth model part, it is very often estimated with certain assumptions about the time course of the means. In the linear LCM-SR, for example, one assumes that observed means change linearly across time. However, we note that it is also possible to estimate the LCM-SR with a saturated mean structure (see Mandys et al., 1994; Wu and West, 2010, for similar suggestions in the LCM context). Which of these specifications is adequate certainly depends on the context, but we think that this issue needs to be addressed in future research, as it is possible to discuss, for example, whether it makes sense to use models with a saturated mean structure to model developmental processes.

## 4. Time-Invariant vs. Time-Varying Covariates

In social science, researchers are often worried about the observed effects being spurious due to some third variable that artificially inflates or deflates the autoregressive or cross-lagged parameters. Thus, a standard procedure is to include covariates in statistical models to investigate whether the observed effects hold above and beyond the influence of these potential confounders. In longitudinal data settings, such covariates can be time-invariant or time-varying.

The effect of these covariates on the variables of interest can be constant across measurement occasions or it can vary across occasions. Time-Invariant Covariates (TICs), to begin with, do not take on different values for the same individual over time. Hence, they only vary between persons. Prominent examples for TICs are gender, ethnicity, country of origin, or birth year. However, the effects of TICs on the key variables in the model can be constant or variable over time. Gender, for instance, can have a different effect on the variables at the first compared to the second time point. Some statistical models, such as the RI-CLPM, control for the effect of observed and *un*observed TICs. However, they assume that the TIC has a constant effect across time, and this is not necessarily the case. Fortunately, at least when the TIC is observed, this assumption can be tested, for example by constraining the effects of the TIC on the key variables to the same value over time and examine if model fit worsens (for an example using change score models, see Johnson et al., 2016). By contrast, in the case of unobserved TICs—that is, TICs that have not been measured—this can neither be modeled nor tested; rather, one must simply make this constant-effect assumption.

The within-person portions of the RI-CLPM and the LCM-SR (and also similar models pertaining to within-person dynamics; Allison, 2009) should be unaffected by the effects of time-constant TICs and should remain the same no matter which and how many time-constant TICs are added to the model. The reason for this is a statistical transformation that is performed in these models. Specifically, the inclusion of random intercepts in the RI-CLPM is akin to a person-mean centering of the included variables (Allison, 2009; Hamaker et al., 2015; Wang and Maxwell, 2015) and a detrending is performed through the inclusion of the LGM portion in the LCM-SR (Curran et al., 2014; Wang and Maxwell, 2015). As a result of centering and detrending, all stable between-person differences— observed and unobserved alike—in demographics, personality, life style, response patterns, method effects, childhood socioeconomic conditions, educational attainment, attractiveness, etc. are removed from the models. Again, this reasoning only holds when the effects of TICs are constant over time, that is, when their effect on the key variables in the model is the same at each measurement occasion (Allison, 2009).

As opposed to TICs, Time-Varying Covariates (TVCs) change across time (Grimm, 2007; Curran and Bauer, 2011). An example of such a variable is marital status, because in a community sample, some people might be married at the onset of the study and over the course of the years some people might divorce, while others might find a (new) partner or might be widowed. Similarly, variables from the personality domain such as self-esteem may be TVCs. Notably, TVCs differ in whether they are affected by previous values of the treatment variable or not. For instance, chronological age may confound the relationship between income at time point *t* and life satisfaction at *t*+1, but it will not be causally influenced by income at *t*−1 (i.e., people do not get older or younger with income). However, a TVC such as marital status may be affected by previous values of the treatment (e.g., income at *t*−1 predicts marital status at time point *t*) and it also affects the key variables measured at the later time point. They are, thus, intermediate variables that lie on the causal path of the key variables measured at the different occasions. Finally, as in the case of TICs, their effect can be constant across time or it can vary with time. Furthermore, when not included into the statistical model (e.g., when they are unobserved), they can affect the parameter estimates in a substantial way, which increases the probability of false interpretations and erroneous conclusions. If observed, TVCs should and can easily be included in all sorts of statistical models in a flexible way so that concurrent and prospective effects of the TVCs on the key variables in the model can be examined (Grimm, 2007; Allison, 2009; Snijders and Bosker, 2012). In the following, we demonstrate the effects of TICs and TVCs for the association between life satisfaction and household income using data from the German Family Panel (Huinink et al., 2011; Brüderl et al., 2019).

## 5. Empirical Illustration: Life Satisfaction and Income

### 5.1. Sample

The data for the present analysis were taken from the first four waves of the representative German Family Panel Pairfam (Huinink et al., 2011; Brüderl et al., 2019). Pairfam is an ongoing study that started in 2008. Initially, 12,402 individuals were interviewed at their homes about a wide variety of demographic, socio-economic, and psychological topics. These participants were re-interviewed each year to also capture changes in their life circumstances (for more information, see https://www.pairfam.de/en).

The analysis for the present study are based on all participants providing at least partial data at the first four measurement occasions. Sample size for the single models ranged from 12,398 to 12,402. Missing data were treated using Full Information Maximum Likelihood (Enders, 2010).

### 5.2. Measures

*Life satisfaction* was measured using a single item (“All in all, how satisfied are you with your life at the moment?”) answered on an 11-point Likert-type rating scale ranging from 0 (*very dissatisfied*) to 10 (*very satisfied*).

Information on *household income* were collected using a single question (“Combining all income types: How much was the total monthly household income for all household members last month?”). We used the log-transformed income variable in the analyses.

We selected *gender* as a time-invariant covariate. Previous research has demonstrated that gender is associated with income in a way that women earn less, even when they occupy similar positions as men (Fields and Wolff, 1995; Gannon et al., 2007). Similarly, gender differences have also been reported for life satisfaction in a way that men report higher satisfaction than women (Koivoumaa-Honkanen et al., 2000).

As a time-varying covariate, we selected individual scores on *self-esteem*, that is, an individual's consideration of him- or herself as a person of worth. Self-esteem has been found to vary both between (Trzesniewski et al., 2003; Kuster and Orth, 2013) and within persons (Mund and Neyer, 2016; Orth et al., 2018). Furthermore, self-esteem has been found to be related to income (Drago, 2011; Orth et al., 2012) and life satisfaction (Diener and Diener, 1995; Mund and Neyer, 2016). In pairfam, self-esteem was measured using three items taken from the Rosenberg self-esteem scale (e.g., “I like myself just the way I am”) answered on a 5-point Likert-type scale ranging from 1 (*does not apply at all*) to 5 (*applies absolutely*). Internal consistency for the scale was adequate in the present sample (ω_*T*1_ = 0.70, ω_*T*2_ = 0.76, ω_*T*3_ = 0.78, and ω_*T*4_ = 0.78, respectively).

### 5.3. Data Analysis

All models were estimated in R 4.0.3 (R Core Team, 2020) using the lavaan package version 0.6-7 (Rosseel, 2012). Full model syntax and complete model outputs can be obtained from https://osf.io/8mvu5/.

To facilitate the interpretation of the model results, we imposed some minor constraints on the parameters. Note that these constraints are not essential for model estimation and can be relaxed in case of bad model fit or for substantive reasons. As shown in Supplementary Table 2 the fit of all models was good. In all models, we constrained the autoregressive and cross-lagged paths to be equal over time. Furthermore, we constrained the residual covariance in all models to be equal over time. Additionally, in the CLPM, we additionally constrained the residual variances to be equal over time; this constraint was not present in the RI-CLPM or the LCM-SR. Without this constraint, the CLPM produced Heywood cases.

In the RI-CLPM and the LCM-SR, we implemented gender, the TIC in the present study, in two ways: First, assuming constant effects over time, we used gender as a predictor of the time-specific observed (RI-CLPM) and latent (LCM-SR) variables but constrained the regression weights to be equal across time. Second, assuming varying effects over time, we allowed the regression weights of gender to vary over time (see https://osf.io/8mvu5/ for the syntax and Mulder and Hamaker, 2021, for a discussion of this approaches). For the CLPM, we conducted a model comparison testing a model with constant effects of the TIC against a model with varying effects of the TIC.

With regard to the TVC (self-esteem), we added the time point-specific scores to the model. This is the simplest way to control for the effects of a TVC and widely-used approach in many research applications (Grimm, 2007). We note, however, that it is also possible to model the TVC in a more complex fashion, for example by fitting a RI-CLPM or a LCM-SR to the TVC as well. However, such strategies differ quite strongly regarding their theoretical rationale, their consequences for the complexity of the model, and their consequences for interpreting the effects.

## 6. Results

Means, standard deviations, and zero-order correlations between all variables are displayed in Table 1. As can be seen, all variables except gender were consistently associated with each other. In Supplementary Table 1, we display the effects of the TIC and the TVC on the key variables in each model. Note that for self-esteem, a method effect is present in the data as the mode of measurement switched from a computer-assisted personal interview to a computer-assisted self-report. This method effect affected mainly the mean scores of self-esteem but not its correlations with other variables (for details, see Mund and Neyer, 2016). Thus, as we are not interested in mean-level changes over time, we left the variable as is.

**Table 1:** Zero-ordecorrelations and descriptive statistics.

	**Zero-order correlations**													
**Variable**	**1**	**2**	**3**	**4**	**5**	**6**	**7**	**8**	**9**	**10**	**11**	**12**	**M**	**SD**
1. T1 satisfaction													7.62	1.75
2. T2 satisfaction	0.50^***^												7.72	1.67
3. T3 satisfaction	0.43^***^	0.55^***^											7.58	1.67
4. T4 satisfaction	0.36^***^	0.44^***^	0.51^***^										7.52	1.71
5. T1 income (Log)	0.18^***^	0.16^***^	0.16^***^	0.15^***^									7.70	0.63
6. T2 income (Log)	0.22^***^	0.25^***^	0.24^***^	0.19^***^	0.63^***^								7.67	0.69
7. T3 income (Log)	0.23^***^	0.27^***^	0.27^***^	0.23^***^	0.61^***^	0.75^***^							7.71	0.65
8. T4 income (Log)	0.22^***^	0.24^***^	0.25^***^	0.24^***^	0.52^***^	0.64^***^	0.77^***^						7.74	0.67
9. Gender	0.00	0.00	-0.01	0.00	-0.04^**^	-0.02	-0.03	-0.04^**^					0.51	0.50
10. T1 Self-esteem	0.41^***^	0.33^***^	0.30^***^	0.25^***^	0.06^***^	0.12^***^	0.12^***^	0.14^***^	-0.13^***^				4.12	0.76
11. T2 Self-esteem	0.31^***^	0.40^***^	0.33^***^	0.28^***^	0.04^*^	0.08^***^	0.10^***^	0.11^***^	-0.13^***^	0.48^***^			3.93	0.86
12. T3 Self-esteem	0.28^***^	0.33^***^	0.43^***^	0.32^***^	0.06^***^	0.10^***^	0.13^***^	0.13^***^	-0.14^***^	0.45^***^	0.54^***^		3.92	0.86
13. T4 Self-esteem	0.27^***^	0.32^***^	0.37*^***^	0.44^***^	0.06^***^	0.09^***^	0.12^***^	0.11^***^	-0.12^***^	0.43^***^	0.49^***^	0.58^***^	3.90	0.84

*M, Mean; SD, Standard Deviation. Gender was coded 0: men, 1: women*.

* *p < 0.05;*

** *p < 0.01;*

*** *p < 0.001*.

In the following, we will discuss the results of the analyses with a particular focus on the comparison between the different specifications of the single models (unconditional models, models including a TIC, and models including a TVC). In comparing the model specifications, we focus on two aspects. First, we inspect whether a given parameter has different levels of statistical significance across the model specifications. Second, we examine whether the parameters from one specification are significantly different from the same parameter in another specification. To this end, we investigate if and to what extent the 95% confidence intervals (CI) of the parameter estimates overlap. If there is no overlap in the CI, the parameters are different from each other at *p* < 0.01, if the proportion overlap is ≤ 0.5 the margin of error, the parameters are different at *p* < 0.05 (Cumming and Finch, 2005).

### 6.1. Cross-Lagged Panel Model

All three models fitted the data well (see Supplementary Table 2); the parameter estimates are displayed in Table 2. We tested whether gender can be considered a TIC with constant effects by comparing a constrained (equal regression weight of gender on all variables) model to an unconstrained model (regression weight estimated freely). The constrained model did not fit worse than the unconstrained model (Δχ^2^ = 4.21, Δ*df* = 6, *p* = 0.649), so we used gender in the present analysis as a TIC with constant effects for the CLPM.

**Table 2:** Comparison of CLPM models.

	**Unconditional**				**Gender (TIC)**				**Self-esteem (TVC)**			
**Parameter**	**EST**	**95% CI**		***p***	**EST**	**95% CI**		***p***	**EST**	**95% CI**		***p***
*Autoregressive Effects*												
a1	0.86	0.83,	0.88	<0.001	0.86	0.83,	0.88	<0.001	0.59	0.57,	0.62	<0.001
a2	0.86	0.84,	0.88	<0.001	0.86	0.84,	0.88	<0.001	0.87	0.85,	0.89	<0.001
*Cross-Lagged Effects*												
c1	0.03	0.02,	0.03	<0.001	0.03	0.02,	0.03	<0.001	0.02	0.01,	0.03	<0.001
c2	0.03	-0.01,	0.06	0.126	0.03	-0.01,	0.06	0.122	0.13	0.10,	0.17	<0.001
*Correlations*												
*r*_*sat*1, *inc*1_	0.28	0.25,	0.32	<0.001	0.28	0.25,	0.32	<0.001	0.23	0.20,	0.26	<0.001
*r*_*satT, incT*_	0.15	0.11,	0.20	<0.001	0.15	0.11,	0.20	<0.001	0.12	0.09,	0.16	<0.001

*TIC, Time-Invariant Covariate; TVC, Time-Varying Covariate. a1 and c1 refer to autoregressive and lagged effects of life satisfaction, respectively, while a2 and c2 refer to the same paths for income. EST: unstandardized regression weight / correlation. 95% CI: lower bound and upper bound of the 95% confidence interval. N= 12,398 for the unconditional model and 12,402 for the model including TIC and TVC*.

As can be seen in Table 2, the parameter estimates of the CLPM were largely consistent and there was a large overlap of the CI, indicating no difference between the parameters. However, there are three exceptions: First, in the model with the TVC (self-esteem), the stability of life satisfaction (path *a*1) was significantly lower (*p* < 0.01) than in the other model specifications. Second, the cross-lagged effect of income on satisfaction (path *c*2), in contrast, was significantly higher (*p* < 0.01) than in the unconditional model and the model with a TIC (gender). Path *c*2 was also statistically significant in the model including a TVC, but not in the other specifications. Third, the overlap of the CI for the initial correlation between life satisfaction and income was less than half the margin of error between the model with a TVC and the other specifications, Thus, the parameter of the model with a TVC is significantly different from the parameter in the two other models (*p* < 0.05). Practically, however, the difference in this correlation is small (*r*_*TVC*_ = 0.23, *r*_*unconditional*/*TIC*_ = 0.28; Δ*r* = 0.05).

### 6.2. Random-Intercept Cross-Lagged Panel Model

**Figure 4:**
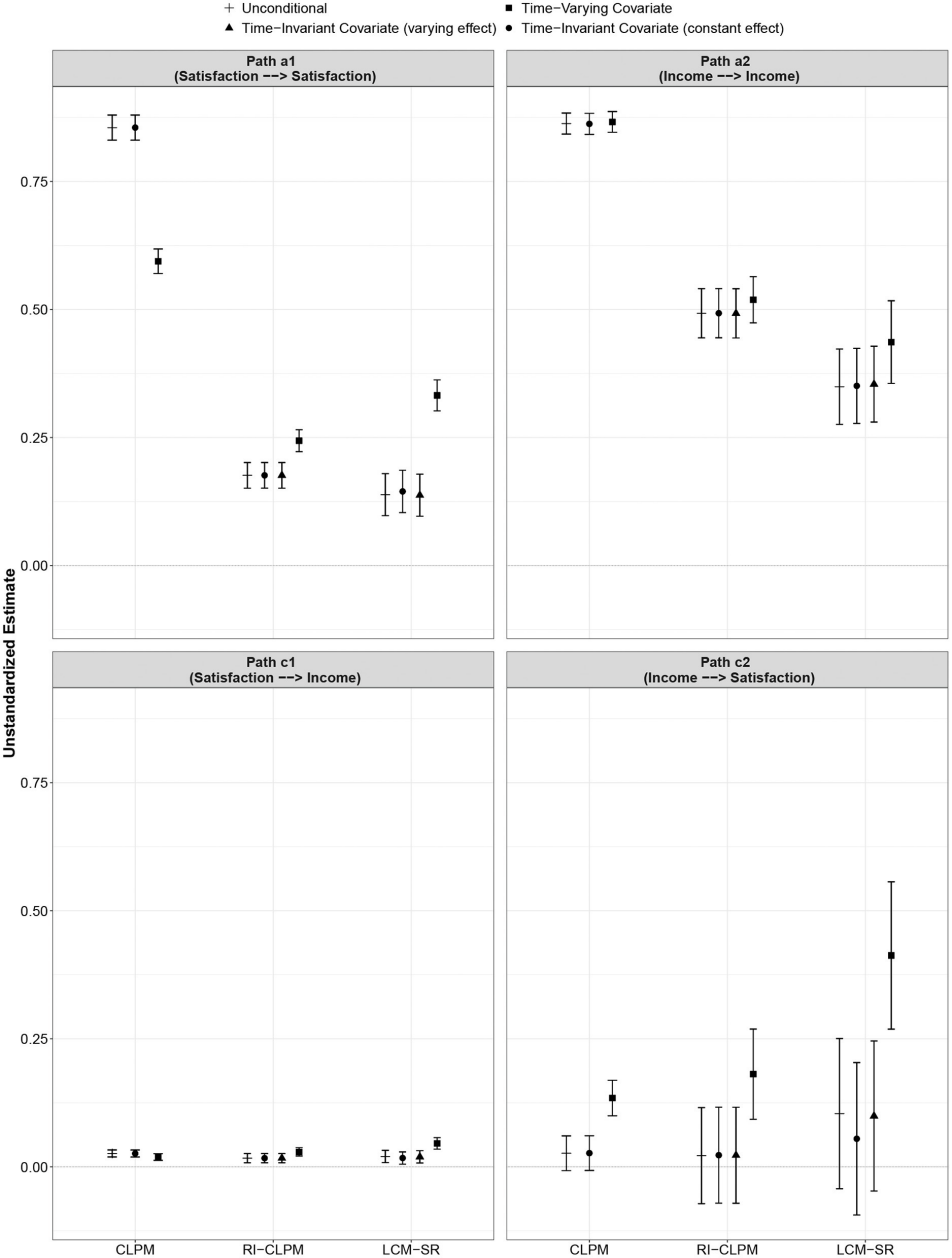
Comparison of autoregressive and cross-lagged parameters across model specifications (unconditional, TIC, TVC) and between models (CLPM, RI-CLPM, LCM-SR). Error bars indicate 95% confidence intervals. Note that income was log-transformed for the analysis so that the scale is different from the scale used to measure life satisfaction. Figure is available under a CC-BY 4.0 license at https://osf.io/8mvu5/.

**Figure 5:**
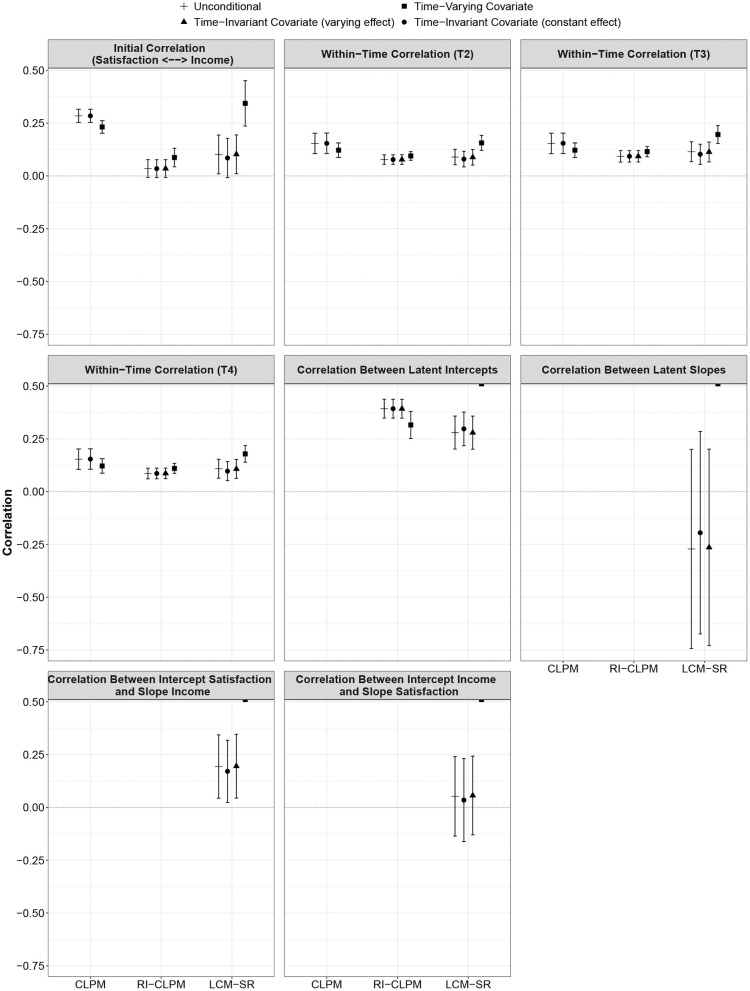
Comparison of correlations across model specifications (unconditional, TIC, TVC) and between models (CLPM, RI-CLPM, LCM-SR). Error bars indicate 95% confidence intervals. Figure is available under a CC-BY 4.0 license at https://osf.io/8mvu5/.

The results for the RI-ClPM model specifications are displayed in Table 3 and in Figures 4, 5. All models fitted the data well (see Supplementary Table 2).

**Table 3:** Comparison of RI-CLPM Models.

	**Unconditional**				**Gender (TIC, constant effect)**				**Gender (TIC, varying effect)**				**Self-esteem (TVC)**			
**Parameter**	**EST**	**95% CI**		***p***	**EST**	**95% CI**		***p***	**EST**	**95% CI**		***p***	**EST**	**95% CI**		***p***
*Autoregressive Effects*																
a1	0.18	0.15,	0.20	<0.001	0.18	0.15,	0.20	<0.001	0.18	0.15,	0.20	<0.001	0.24	0.22,	0.27	<0.001
a2	0.49	0.44,	0.54	<0.001	0.49	0.44,	0.54	<0.001	0.49	0.44,	0.54	<0.001	0.52	0.47,	0.56	<0.001
*Cross-Lagged Effects*																
c1	0.02	0.01,	0.03	<0.001	0.02	0.01,	0.03	<0.001	0.02	0.01,	0.03	<0.001	0.03	0.02,	0.04	<0.001
c2	0.02	-0.07,	0.12	0.648	0.02	-0.07,	0.12	0.634	0.02	-0.07,	0.12	0.636	0.18	0.09,	0.27	<0.001
*Correlations*																
*r*_*Ix, Iy*_	0.39	0.35,	0.44	<0.001	0.39	0.35,	0.44	<0.001	0.39	0.35,	0.44	<0.001	0.32	0.25,	0.38	<0.001
*r*_*sat*1, *inc*1_	0.03	-0.01,	0.08	0.105	0.03	-0.01,	0.08	0.109	0.03	-0.01,	0.08	0.105	0.09	0.04,	0.13	<0.001
*r*_*sat*2, *inc*2_	0.08	0.05,	0.10	<0.001	0.08	0.05,	0.10	<0.001	0.08	0.05,	0.10	<0.001	0.09	0.07,	0.12	<0.001
*r*_*sat*3, *inc*3_	0.09	0.07,	0.12	<0.001	0.09	0.07,	0.12	<0.001	0.09	0.07,	0.12	<0.001	0.12	0.09,	0.14	<0.001
*r*_*sat*4, *inc*4_	0.09	0.06,	0.11	<0.001	0.09	0.06,	0.11	<0.001	0.09	0.06,	0.11	<0.001	0.11	0.09,	0.13	<0.001

*TIC, Time-Invariant Covariate; TVC, Time-Varying Covariate. a1 and c1 refer to autoregressive and lagged effects of life satisfaction, respectively, while a2 and c2 refer to the same paths for income. EST: unstandardized regression weight / correlation. 95% CI: lower bound and upper bound of the 95% confidence interval. N= 12,398 for the unconditional model and 12,402 for the model including TIC and TVC*.

As with the CLPM, the parameter estimates were largely consistent across the four specifications. Again, only the parameters from the model incorporating self-esteem as a TVC differed from the other parameter estimates in some instances. The parameter *a*1, the within-person stability of life satisfaction, was significantly higher when incorporating a TVC as compared to all other models (*p* < 0.01). The parameter *c*2, the within-person effect of deviations from the person-specific mean in income on future deviations from the person-specific mean in life satisfaction, was higher in the model with a TVC than in all other models (*p* < 0.05). With regard to the initial correlation between life satisfaction and income, the parameters of all model specifications were equivalent; however, in the model with a TVC, this correlation reached statistical significance whereas it was not statistically significant in the other specifications.

### 6.3. Latent Curve Model With Structured Residuals

The results for the LCM-SR are displayed in Table 4 and in Figures 4, 5. Model fit of all four specifications was good (see Supplementary Table 2). However, in the model with self-esteem as a TVC, the variance of the intercept and the slope of life satisfaction was negative and statistically significant. Accordingly, we only interpret the results of this model for illustrative purposes; in specific research applications, a model that produces inadmissible parameter estimates should not be interpreted.

**Table 4:** Comparison of LCM-SR Models.

	**Unconditional**				**Gender (TIC, constant effect)**				**Gender (TIC, varying effect)**				**Self-esteem (TVC)*^a^***			
**Parameter**	**EST**	**95% CI**		***p***	**EST**	**95% CI**		***p***	**EST**	**95% CI**		***p***	**EST**	**95% CI**		***p***
*Autoregressive Effects*																
a1	0.14	0.10,	0.18	<0.001	0.14	0.10,	0.19	<0.001	0.14	0.10,	0.18	<0.001	0.33	0.30,	0.36	<0.001
a2	0.35	0.28,	0.42	<0.001	0.35	0.28,	0.42	<0.001	0.35	0.28,	0.43	<0.001	0.44	0.36,	0.52	<0.001
*Cross-Lagged Effects*																
c1	0.02	0.01,	0.03	0.001	0.02	0.01,	0.03	0.005	0.02	0.01,	0.03	0.002	0.05	0.03,	0.06	<0.001
c2	0.10	-0.04,	0.25	0.165	0.05	-0.09,	0.20	0.471	0.10	-0.05,	0.25	0.184	0.41	0.27,	0.56	<0.001
*Correlations*																
*r*_*Ix, Iy*_	0.28	0.20,	0.36	<0.001	0.30	0.22,	0.38	<0.001	0.28	0.20,	0.36	<0.001	—	—	—	—
*r*_*Sx, Sy*_	-0.27	-0.74,	0.20	0.259	-0.19	-0.67,	0.28	0.426	-0.26	-0.73,	0.20	0.266	—	—	—	—
*r*_*Ix, Sy*_	0.19	0.04,	0.34	0.011	0.17	0.02,	0.32	0.023	0.20	0.04,	0.35	0.011	—	—	—	—
*r*_*Iy, Sx*_	0.05	-0.14,	0.24	0.583	0.03	-0.16,	0.23	0.731	0.06	-0.13,	0.24	0.554	—	—	—	—
*r*_*sat*1, *inc*1_	0.10	0.01,	0.19	0.030	0.08	-0.01,	0.18	0.073	0.10	0.01,	0.19	0.029	0.34	0.24,	0.45	<0.001
*r*_*sat*2, *inc*2_	0.09	0.05,	0.13	<0.001	0.08	0.04,	0.12	<0.001	0.09	0.05,	0.13	<0.001	0.16	0.12,	0.19	<0.001
*r*_*sat*3, *inc*3_	0.11	0.07,	0.16	<0.001	0.10	0.06,	0.15	<0.001	0.11	0.07,	0.16	<0.001	0.20	0.15,	0.24	<0.001
*r*_*sat*4, *inc*4_	0.11	0.06,	0.15	<0.001	0.10	0.05,	0.14	<0.001	0.11	0.06,	0.15	<0.001	0.18	0.14,	0.22	<0.001

a *The model produced a statistically significant negative variance for the intercept and the slope of life satisfaction. Results need to be interpreted with caution. TIC, Time-Invariant Covariate; TVC, Time-Varying Covariate. a1 and c1 refer to autoregressive and lagged effects of life satisfaction, respectively, while a2 and c2 refer to the same paths for income. EST: unstandardized regression weight / correlation. 95% CI: lower bound and upper bound of the 95% confidence interval. N= 12,398 for the unconditional model and 12,402 for the model including TIC and TVC*.

The parameter *a*1 estimated in the model with TVC was significantly different from *a*1 as estimated in the other specifications (*p* < 0.01). Furthermore, the cross-lagged paths *c*1 (*p* < 0.05) and *c*2 (*p* < 0.01) differed between the model with TVC and all other models. Furthermore, path *c*2 only reached statistical significance in the model with TVC, whereas it was not statistically significant in any of the other specifications.

With regard to correlations, the model with TVC yielded a significantly higher estimate of the initial association between life satisfaction and income than the other specifications (*p* < 0.01). In the model including gender as a TIC with constant effects, the initial correlation was not statistically significant, as opposed to all other specifications. However, the parameter was not different from the parameters estimated in the unconditional LCM-SR and the model in which gender was incorporated as a TIC with varying effects. No differences between model specifications were found neither regarding the correlations between the intercept of life satisfaction and the slope of income nor the correlation between the intercept of income and the slope of life satisfaction. The time point-specific correlations between deviations in life satisfaction and income were higher in model incorporating a TVC. At all measurement occasions, the estimate from this model was significantly different from the parameters estimated in the other specifications (proportion overlap between 0.00 and 0.25; *p* < 0.05).

## 7. Discussion

In the present study, we examined changes in the precision and interpretation of parameter estimates (in terms of statistical significance) across different specifications of the CLPM, RI-CLPM, and LCM-SR—three prominent models developed to investigate reciprocal influences between at least two constructs. More specifically, using the example of reciprocal effects between life satisfaction and income, we investigated the robustness of parameters across an unconditional model (no covariates), a model including a time-invariant covariate with constant or varying effects (gender), and a time-varying covariate (self-esteem). Across all models and specifications, we found evidence for satisfaction being associated with future income, whereas the reverse path from income to satisfaction was only statistically significant in the models including a TVC. Although we used a very simple setup, we believe that this minimalist approach is still informative. Gender is a standard covariate included in many applications and self-esteem is a construct with well-documented associations with both life satisfaction and income (Diener and Diener, 1995; Drago, 2011; Orth et al., 2012; Mund and Neyer, 2016).

In the present study, we used self-esteem as a TVC. Given prior research (DeNeve and Cooper, 1998; Lyubomirsky et al., 2005; Drago, 2011; Orth et al., 2012; Mund and Neyer, 2016), it could be assumed that self-esteem affects life satisfaction and income and does not operate as a collider, which is also supported by the patterns of correlation (see Table 1) and the main effects of self-esteem on other variables in the model (Supplementary Table 1). Thus, the model using self-esteem as a TVC seems plausible. In addition, we believe that the unconditional model is similarly plausible. Gender, which was used as a TIC in the present study, showed stable effects on life satisfaction and income over time (see Supplementary Table 1) and such time-constant effects of TICs are already considered in the RI-CLPM and the LCM-SR.

The results of the present study are in line with other studies showing that the different models can arrive at different conclusions (Hounkpatin et al., 2018; Mund and Nestler, 2019; Orth et al., 2021). In the present study, this difference was particularly prominent for the autoregressive effects (see Figure 4), which were significantly stronger in the CLPM than in the RI-CLPM and the LCM-SR. The results of the RI-CLPM and the LCM-SR were largely consistent with the exception that the estimate for path *a*2 (autoregressive effect of income) was stronger in the RI-CLPM and that the initial correlation between satisfaction and income was not statistically significant in three of the four RI-CLPM specifications, but in only one of the four LCM-SR specifications.

In terms of model comparison, albeit not the main focus of the present manuscript, although all models fitted the data quite well, a particular good fit was observed for the CLPM across all model specifications (see also Orth et al., 2021). This observation stresses the importance of selecting a model that suits the research question at hand. Although the RI-CLPM and the LCM-SR fit slightly worse than the CLPM in the present study and are more complex in terms of computation and interpretation, they also offer the opportunity to address research questions pertaining to within-person dynamics—which is not possible with the CLPM (Hamaker et al., 2015; Berry and Willoughby, 2017).

The main focus of the present study was the comparison of parameter estimates across different model specifications (unconditional, TIC, TVC). Regarding this comparison, we found that the parameter estimates of the CLPM, RI-CLPM, and LCM-SR were largely invariant when a TIC was included. The parameter estimates remained virtually identical and there was only one case where the parameters differed in their *p*-value. Specifically, the initial correlation between life satisfaction and income was not statistically significant in the LCM-SR including gender as a TIC with constant effects, whereas it was statistically significant in the other specifications. It should be noted, however, that the association between gender and both life satisfaction and income was surprisingly low in this data set. TICs with stronger associations with the key variables might lead to notable shifts in the parameter estimates. It should be noted, though, that these results might not generalize to other contexts and data (Simons et al., 2017), and that there might be cases where, for instance, also TICs might heavily influence parameter estimation.

All models turned out to be sensitive to the inclusion of a TVC. Some key parameters of the models changed markedly when including the TVC. Most prominently, the parameter *a*1 (satisfaction → satisfaction) decreased in the CLPM but increased in the RI-CLPM and the LCM-SR. Furthermore, the parameter *c*2 (income → satisfaction) increased in all three models and became statistically significant. The LCM-SR was affected most strongly in this regard as the inclusion of self-esteem, the TVC in this analysis, led to inadmissible parameter estimates and, thus, a barely interpretable model. It is difficult to track down the exact point where the model estimation encountered problems. The data set is large and has been used already for complex models including dyadic cross-lagged panel models (Johnson et al., 2017), latent change score models (Mund et al., 2015; Johnson et al., 2016), and growth mixture models (Mund and Neyer, 2016). However, other studies have shown that the LCM-SR is more prone than the CLPM and the RI-CLPM to run into estimation problems (Orth et al., 2021; Scott, 2021).

The changes in the model parameters following the inclusion of a TVC do not necessarily mean that these parameters are biased or not trustworthy (except in the LCM-SR, where larger estimation issues occurred). The inclusion of a TVC is supposed to better approximate a true causal effect (Allison, 2009). Whether this approximation is successful or not depends on the assumptions underlying the specific model and its fit to the true causal model (Scott, 2021)—that is unknown in most research scenarios. If the underlying assumptions are not fulfilled and the assumed TVC is, in fact, a collider (i.e., a variable that is only affected by but not itself affecting the key variables in the model), the resulting parameter estimates might be biased (Elwert and Winship, 2014).

Whether to include a TVC or not in a given analysis can be difficult to decide beforehand, as there might be multiple plausible causal models (Rohrer and Lucas, 2020). In such cases, it might be worthwhile to consider and contrast a range of plausible model specifications (Del Giudice and Gangestad, 2021) and to make explicit the assumptions underlying these models using a directed acyclic graph (Elwert and Winship, 2014) or matrices of implied causation (Brick and Bailey, 2020). Based on these explicit causal assumptions, researchers can take an informed decision and argue for the theoretically most meaningful model and flesh out the causal chains (Rohrer and Lucas, 2020).

Taken together, the present case study underscores the importance of both careful model checking and transparent reporting of results. It is well-known that the inclusion or exclusion of covariates can have serious consequences for the interpretation of results (Simmons et al., 2011). As a consequence, researchers should report or document all model results with and without covariates included (see also Simmons et al., 2011; Asendorpf et al., 2013). Finally, when possible, as many assumptions as possible about the variables included in a model should be tested (Elwert and Winship, 2014; Brick and Bailey, 2020). For example, whether an observed TIC has constant or varying effects is a straightforward assumption to test (Johnson et al., 2016; Mulder and Hamaker, 2021). Such tests can safeguard researchers against possible imprecise or shifting parameter estimates and, hence, erroneous conclusions.

### 7.1. Limitations

As limitations, we note the minimalist setup of the present study with only one TIC and only one TVC, and both were weakly to moderately related to the key variables in the model. The present study should, thus, be considered a case study. In most applications, researchers might wish to include even more covariates such as age, health, psychological functioning, occupational status, and many more. The fact that we found shifts in parameter estimates even in this minimalist and not atypical setup underscores the importance of carefully checking and comparing different model specifications.

We also stress that we cannot evaluate the model specifications with regard to their capability to discover the true effect or generalize the findings far beyond the present context. To achieve these goals, simulation studies that explicitly examine the performance of the models under a wider variety of circumstances might be a worthwhile endeavor (Scott, 2021). However, we note that particularly the LCM-SR has been reported before to run into estimation issues more easily than the RI-CLPM and the CLPM (Orth et al., 2021).

Finally, we investigated only one method to incorporate TVCs. This method is easily accessible to most researchers and a de facto standard to adjust for TVC (Grimm, 2007; Mulder and Hamaker, 2021). Alternative specifications are possible (Curran and Bauer, 2011), but such alternatives come with specific challenges and assumptions. For instance, self-esteem could have been modeled as a third developmental process in the LCM-SR, but it is then difficult to decide which process is primary and which secondary. Furthermore, extending the models in this way would increase complexity and might increase the risk of convergence issues. Future (simulation) studies might pay attention to differences in incorporating TVCs to equip researchers with guidelines how to decide for a specific implementation.

Taken together, we used data from Germany to examine the association between life satisfaction and income. Several general constraints on generalizability need to be acknowledged regarding the main results and the sample in general (Simons et al., 2017). For example, the general pattern of results might be different in samples from different countries or cultures, or even when sampling individuals with lower socio-economic status. Regarding the research question, we stress again that other research contexts might yield different results. For example, it might be conceivable that the inclusion of a TIC can be accompanied by large shifts in the parameter estimates, whereas the inclusion of a TVC leaves the estimates unaffected. Thus, the inclusion or exclusion of covariates and how they are modeled best needs to be considered carefully for each research question (Elwert and Winship, 2014; Rohrer and Lucas, 2020).

### 7.2. Conclusion

In the present study, we examined the influences of time-invariant and time-varying covariates on the parameter estimates of three popular models for investigating reciprocal influences between two or more variables over time—the CLPM, RI-CLPM, and LCM-SR. We found that particularly the inclusion of time-varying covariates were associated with changes in the parameter estimates. Although it is plausible and has been demonstrated repeatedly that the inclusion of additional variables in a model might change parameter estimates and their interpretation, the present study extends these findings to recently developed models such as the RI-CLPM and the LCM-SR. These results are important, because models that separate within- from between-person variance have sometimes been considered models capable of uncovering causal associations (Allison, 2009). In light of shifts in parameter estimates, this notion might need to be taken with some caution. The results of the present study rather underscore the necessity of building statistical models ideally based on a strong theory that clearly defines the role of all included variables in the causal process (Rohrer and Lucas, 2020; Del Giudice and Gangestad, 2021). In cases where such a strong theory is not available, researchers still need to be explicit about why certain variables are (not) included in the model and which role they play in the assumed process (Elwert and Winship, 2014; Brick and Bailey, 2020; Rohrer and Lucas, 2020).

## Data Availability Statement

Publicly available datasets were analyzed in this study. This data can be found here: Gesis Data Archive https://doi.org/10.4232/pairfam.5678.10.0.0.

## Ethics Statement

Ethical review and approval was not required for the study on human participants in accordance with the local legislation and institutional requirements. The patients/participants provided their written informed consent to participate in this study.

## Author Contributions

MM, MJ, and SN conceived the study. MM analyzed the data and drafted the manuscript. MJ and SN reviewed and revised the manuscript. All authors contributed to the article and approved the submitted version.

## Conflict of Interest

The authors declare that the research was conducted in the absence of any commercial or financial relationships that could be construed as a potential conflict of interest.

## Publisher's Note

All claims expressed in this article are solely those of the authors and do not necessarily represent those of their affiliated organizations, or those of the publisher, the editors and the reviewers. Any product that may be evaluated in this article, or claim that may be made by its manufacturer, is not guaranteed or endorsed by the publisher.
