# Supplementary material for: Changes in Size and Interpretation of Parameter Estimates in Within-Person Models in the Presence of Time-Invariant and Time-Varying Covariates
Source: Front Psychol. 2021 Sep 1;12:666928. doi: 10.3389/fpsyg.2021.666928 (PMC8441132; doi:10.3389/fpsyg.2021.666928)
Supplement: Supplementary file 1 [file Table_1.PDF]

Supplementary Material

Table S1. Effects of TIC and TVC on Main Variables

	Gender (TIC)				Self-Esteem (TVC)			
Parameter	EST	95% CI		*p*	EST	95% CI		*p*
				Cross-Lagged Panel Model				
Effects on Life Satisfaction								
T1	0.01	–0.06,	0.07	.179	0.93	0.89,	0.96	<.001
T2	0.01	–0.06,	0.07	.868	0.44	0.40,	0.47	<.001
T3	–0.03	–0.09,	0.04	.452	0.44	0.40,	0.48	<.001
T4	0.06	–0.02,	0.13	.123	0.48	0.44,	0.52	<.001
Effects on Income								
T1	–0.03	–0.06,	0.00	.073	0.08	0.06,	0.10	<.001
T2	–0.17	–0.05,	0.01	.264	0.01	–0.01,	0.02	.545
T3	0.00	–0.03,	0.03	.983	0.03	0.01,	0.04	.001
T4	–0.02	–0.05,	0.01	.176	0.01	–0.01,	0.02	.507
				Random-Intercept Cross-Lagged Panel Model				
Effects on Life Satisfaction								
T1	0.01	–0.06,	0.07	.858	0.89	0.85,	0.93	<.001
T2	0.01	–0.06,	0.08	.795	0.57	0.54,	0.61	<.001
T3	–0.03	–0.09,	0.05	.568	0.62	0.58,	0.65	<.001
T4	0.04	–0.04,	0.12	.307	0.63	0.59,	0.67	<.001
Effects on Income								
T1	–0.03	–0.06,	0.00	.059	0.06	0.04,	0.09	<.001
T2	–0.04	–0.08,	–0.01	.014	0.02	–0.00,	0.03	.080
T3	–0.04	–0.07,	0.00	.036	0.03	0.02,	0.05	<.001
T4	–0.05	–0.09,	–0.02	.004	0.02	0.01,	0.04	.012
				Autoregressive Latent Trajectory Model with Structured Residuals				
Effects on Life Satisfaction								
T1	–0.02	–0.08,	0.04	.429	0.89	0.86,	0.93	<.001
T2	0.07	0.02,	0.11	.004	0.61	0.58,	0.63	<.001
T3	0.00	–0.05,	0.05	.899	0.56	0.54,	0.59	<.001
T4	0.01	–0.06,	0.07	.873	0.55	0.52,	0.56	<.001
Effects on Income								
T1	–0.03	–0.06,	0.00	.077	0.08	0.06,	0.10	<.001
T2	–0.03	–0.05,	–0.01	.001	0.01	0.00,	0.02	.047
T3	–0.03	–0.05,	–0.01	.006	0.02	0.01,	0.03	.001
T4	–0.03	–0.06,	–0.01	.019	0.03	0.01,	0.40	<.001

Note. $N = 12{,}402$. EST: unstandardized regression weight / correlation. 95% CI: lower bound and upper bound of the 95% confidence interval. TIC: Time-Invariant Confounder. TVC: Time-Varying Confounder.

Table S2. Summary of Model Fit

Model	χ^2	df	p	CFI	RMSEA	SRMR	AIC	BIC
Cross-Lagged Panel Model								
Unconditional	34.096	18	.012	0.999	0.008	0.011	164 551.282	164 744.339
varying TIC	33.999	18	.013	0.999	0.008	0.010	182 548.215	182 815.537
constant TIC	38.205	24	.033	0.999	0.007	0.011	182 540.420	182 763.189
TVC[a]	732.137	42	<.001	0.978	0.036	0.039	240 110.346	240 466.764
Random-Intercept Cross-Lagged Panel Model								
Unconditional	79.853	19	<.001	0.996	0.016	0.016	164 595.039	164 780.672
varying TIC	79.235	19	<.001	0.996	0.016	0.014	182 591.450	182 851.347
constant TIC	83.608	25	<.001	0.997	0.014	0.014	182 583.823	182 799.166
TVC	706.485	43	<.001	0.979	0.035	0.049	240 082.695	240 431.687
Autoregressive Latent Trajectory Model with Structured Residuals								
Unconditional	116.986	16	<.001	0.994	0.023	0.015	164 638.172	164 846.080
varying TIC	50.368	16	<.001	0.998	0.013	0.011	182 568.584	182 850.757
constant TIC	114.324	22	<.001	0.995	0.018	0.014	182 620.540	182 858.159
TVC	633.429	40	<.001	0.981	0.035	0.041	240 015.638	240 386.907

Note. df: degrees of freedom for the χ^2-test. p: p-value of the χ^2-test. CFI: Comparative Fit Index. RMSEA: Root Mean Square Error of Approximation. SRMR: Standardized Root Mean Square Residual. AIC: Akaike Information Criterion. BIC: Bayesian Information Criterion. [a]: For the CLPM with TVC, the lavaan package threw a warning that the optimizer could not find a solution. Inspecting the model results did not yield any obvious mistakes or improper parameter estimates, which is why we decided to keep the model.
